# An *in vitro* bladder model with physiological dynamics: Vesicoureteral reflux alters stent encrustation pattern

**DOI:** 10.3389/fbioe.2022.1028325

**Published:** 2022-10-11

**Authors:** Shaokai Zheng, Pedro Amado, Dominik Obrist, Fiona Burkhard, Francesco Clavica

**Affiliations:** ^1^ ARTORG Center for Biomedical Engineering Research, University of Bern, Bern, Switzerland; ^2^ Department of Urology, Inselspital, Bern University Hospital, University of Bern, Bern, Switzerland

**Keywords:** urology, stent, encrustation, bladder, obstruction, reflux, stone, urodynamics

## Abstract

*In vitro* models are indispensable to study the physio-mechanical characteristics of the urinary tract and to evaluate ureteral stent performances. Yet previous models mimicking the urinary bladder have been limited to static or complicated systems. In this study, we designed a simple *in vitro* bladder model to simulate the dynamics of filling and voiding. The physio-mechanical condition of the model was verified using a pressure-flow test with different bladder outlet obstruction levels, and a reflux test was performed to qualitatively demonstrate the stent associated vesicoureteral reflux (VUR). Finally, the setup was applied with and without the bladder model to perform encrustation tests with artificial urine on commercially available double-J stents, and the volumes of luminal encrustations were quantified using micro-Computed Tomography and image segmentation. Our results suggest that, VUR is an important factor contributing to the dynamics in the upper urinary tract with indwelling stents, especially in patients with higher bladder outlet obstruction levels. The influence of VUR should be properly addressed in future *in vitro* studies and clinical analyses.

## 1 Introduction

Double-J ureteral stents have been widely used in urology to treat urinary tract obstructions since their invention (Finney, 1978). Despite numerous upgrades in materials, surface treatment, and structural design, encrustation remains one of the primary complications of indwelling stents. Here, we refer to encrustation as the collective volume of conditioning films, crystalline particle depositions, and biofilms on the stent surface. Such aggregates grow over time, creating occlusions in the stent lumen or side holes (Yoshida et al., 2021; Zheng et al., 2021), which eventually lead to secondary obstruction of the upper tract. In case of infectious stones such as struvite, the bacteria in the encrustation also increases the risk of urinary tract infection (Hobbs et al., 2018).

Many efforts have been made to understand and hinder the encrustation process. *In vitro* experiments with artificial urine (AU) have successfully revealed the roles of biofilm (Hobbs et al., 2018), stent indwelling time (Cauda et al., 2017), and stent design parameters (Mosayyebi et al., 2018) in the dynamic process. Most of these studies incorporate a unidirectional flow through the ureter to mimic physiological flow conditions during bladder filling. The cyclic changes of the physio-mechanical conditions in the urinary tract during filling and voiding were often omitted.

Since the placement of a stent prevents closure of the ureterovesical junction (UVJ), and reduces or stops ureteral peristalsis (Mosli Hisham et al., 1991), the rapid increase in intravesical pressure during voiding creates an adverse pressure gradient that results in a retrograde ascension of urine, referred to as vesicoureteral reflux (VUR). According to a recent study using porcine models with indwelling stents (de la Cruz et al., 2021), 91.7% of the test subjects experienced VUR. Even though the stent induced VUR mainly affected the distal ureter, and was mostly classified as low grade according to the classification of the International Reflux Study Committee (de la Cruz et al., 2021), its impact on the encrustation process was not discussed and remained unclear.

On the one hand, the retrograde flux of urine assists the ascension of bacteria that exacerbates biofilm formation (Scotland et al., 2019). On the other hand, the kinetic energy of VUR might have a flushing effect that removes some of the crystalline particle depositions. Knowledge of the mechanism may be used to optimize stent selection in patients and to benefit future stent designs.

The current study was carried out in three steps. First, we designed an active bladder model and validated it using a pressure-flow test to reproduce human physiological and pathological conditions. Second, a reflux test was performed to qualitatively examine the reflux level induced by the bladder model. Finally, clean commercially available double-J stents were placed in a ureter model with supersaturated AU with and without the bladder model. The amount of encrustations on stents collected from these experiments was analyzed by micro-Computed Tomography (µ-CT) and image segmentation. As such, encrustation patterns on the stents were evaluated with respect to VUR. We found that stent associated VUR may play an important role, especially in patients with bladder outlet obstruction (BOO), and therefore should be carefully addressed, whether in basic research or in the evaluation of new stent designs.

## 2 Materials and methods

### 2.1 *In vitro* model

**FIGURE 1:**
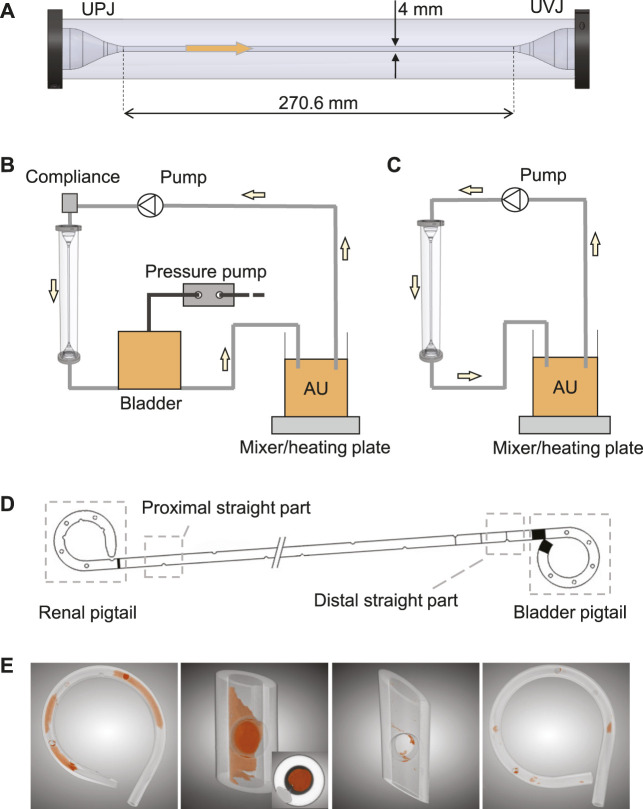
**(A)** The ureter model with conical ends to simulate UPJ and UVJ. The arrow shows the flow direction. Schematics of the complete *in vitro* setup with and without the bladder model are shown in **(B)** and **(C)**, respectively. **(D)** Illustration of the four sections of a stent chosen for this study. **(E)** Examples of the segmented images highlighting the luminal encrustations. The inset in the second image shows a completely occluded stent lumen.

The *in vitro* model consisted of three components, the ureter model (Zheng et al., 2022a), the bladder model, and the urethra model. The ureter model was made by silicone molding, and was 270.6 mm long with a 4 mm inner diameter, including two conical ends to simulate the ureteropelvic junction (UPJ) and UVJ (Figure 1A). Since ureteral peristalsis is known to be significantly reduced or completely inhibited by indwelling stents (Mosli Hisham et al., 1991), the rigidity of the ureter model was deemed appropriate for the purpose of this study. The bladder was modeled with a cylindrical vessel made of acrylic glass. Its capacity was 500 ml, and the maximum fluid volume during experiments was limited to 480 ml, which was similar to the normal volume of human bladder (Zheng et al., 2021). The dynamics of the bladder was realized by connecting a pressure pump (OB1 MK3+, Elvesys Group, FR) to the bladder model, allowing programmable pressure waveforms. The waveform was used to simulate the pressure condition in the human bladder during the cyclic phases of filling and voiding. The urethra model in this study was simplified as a silicone tube with a circular cross section of 6 mm inner diameter, connected to the outlet of the bladder model to mimic the urethra during voiding.

In this study, three types of tests were designed using different combinations of these models. First, the bladder and urethra models were used to perform the pressure-flow tests to validate the urodynamic characteristics of the *in vitro* setup. Second, all models were connected to simulate the full urinary tract, and the reflux test was carried out to verify the presence of reflux in the ureter model. Finally, two encrustation tests with AU were run with and without the bladder model, respectively, to evaluate the impact of reflux on the encrustation process. The details of each test are given below.

### 2.2 Pressure-flow test

The pressure-flow test was performed with water to validate the control protocol. To do this, the inlet of the bladder model was closed, simulating a closed UVJ during voiding without an indwelling stent. The bladder model was manually filled up to 480 ml, and outlet at the end of the urethra model was kept at the same height to avoid outflow. To initiate the voiding phase, the pressure pump was used to increase the pressure level in the bladder model, and the water was expelled through the urethra model.

To measure the pressure during voiding, a 2Fr Millar pressure catheter (SPR-320NR, Millar Inc., Houston, TX, United States) was inserted through the urethra model, reaching to the bottom of the bladder model. The fluid expelled through the urethra model was weighed using a scale (NV511, OHAUS Corporation, United States), and the data was recorded by a computer, where the instantaneous flow rate was calculated.

To explore the potential of the bladder model to mimic patho-physiological conditions, the outlet cross-sectional area of the urethra model was tested with 0%, 75%, and 88% occlusion to simulate the unobstructed, equivocal, and obstructed bladder outlet, respectively, as defined in (Abrams, 1999). The two occluded states were realized by connecting smaller tubes with 3 mm and 2 mm inner diameter, respectively, to the original urethra model through a barbed connector. The same pressure pump protocol was applied for all tests, which produced similar pressure waveforms as in (Abrams, 2006). The exact shape can be found in the Supplementary Material with further explanations on the implementation. The pressure, flow rate, and residual volume for each case were measured and compared. The residual volume was calculated by subtracting the voided volume from the starting volume (480 ml) of fluid in the bladder, which was kept the same for all tests presented in this study.

### 2.3 Reflux test

To evaluate the reflux level induced by the current setup, the ureter model with an indwelling stent, a 6Fr/26 cm double-J polyurethane stent (PURE Medical Device SA, CH), was connected to the bladder model, which was filled with premixed dye color as tracing agent. Since all components of the model were rigid, to allow reflux during voiding, a three-way valve was connected to the inlet of the ureter model and to a syringe filled with approximately 53 ml of air. The same voiding protocol from the pressure-flow test was used with two of the obstruction levels 0% and 88%, as described in Section 2.2, to demonstrate the impact of BOO. During the test, the lower part of the ureter was filmed during the voiding phase to observe the retrograde ascension of the dye. The distance traveled by the dye was recorded and compared. Note that, we do not assume any causal relationship between stent placement and BOO, but rather aim to test the reflux level under the influence of obstructive lower urinary tract symptoms, which might be of interest to some audience.

### 2.4 Encrustation test

After validating the pressure-flow condition and the presence of reflux, encrustation tests were performed with and without the bladder model (Figures 1B,C) to simulate the urinary flow condition with and without reflux, respectively. Both experiments were run with supersaturated AU (Mosayyebi et al., 2018). The AU was prepared 48 h prior to the start of the experiment and was kept at 37°C throughout the running time on a heating plate incorporated with magnetic mixer (MR 3001K, Heidolph Instruments, DE) stirring at 150 rpm. The initial pH of AU was seven and was adjusted every 24 h with diluted hydrochloric acid (HCl). The range of pH during all experiments was 6–8.

For both experiments, the filling phase was driven by a peristaltic pump (LabN1 with YZ1515x pump head, Shenchen Precision Pump Co. Ltd., CN) at 2 ml/min from the reservoir into the ureter model. The same voiding protocol from the pressure-flow test without obstructing the urethra model was used, and was programmed to trigger every 4 h, giving a pre-voiding urine volume of 480 ml.

Clean stents with the same features as those in the reflux test were used. Each stent was inserted into the ureter model, and the flow was kept running for 10 days. The resulting encrusted stents were collected and analyzed using the methods reported in (Zheng et al., 2022b), which is briefly described as follows.

After being retrieved from the ureter model, each stent was dried in an oven at 60°C for 3 h to remove residual urine, which helped solidify the sample and prevent contamination during the following analyses. Since the µ-CT scan was done in batches, the actual days between stent removal and date of analysis varied between stent samples. Therefore, drying the sample before cutting actually helped improving the consistency of wetness between samples, reducing further uncertainties during sample handling. Four sections of each stent were cut and isolated, i.e., the renal pigtail, the proximal straight section near the UPJ, the distal straight section near the UVJ, and the bladder pigtail (Figure 1D). Since the two junctions are the beginning and the end of the ureter, comparing the level of encrustation between these two locations was of interest, especially in the presence of VUR. To avoid the uncertainty of encrustations flaking off at the cut site, the cutlines were chosen sufficiently far away from the regions of interest, and the procedure was done carefully using a tweezer to prevent movement of the stent sample during cutting.

To derive the encrustation volumes, each section of the stent was scanned using a µ-CT scanner (SCANCO Medical AG, Bruettisellen, CH), and the acquired images were segmented using a deep learning model known as the U-Net (Ronneberger et al., 2015) implemented in the Dragonfly software (v2020.1, Object Research Systems Inc., CA). Examples of the segmentation results are shown in Figure 1E, where the spatial distribution of the encrustation is preserved.

For this study, we only extracted the luminal encrustations (including the encrustations in the side holes) because encrustations on the external surfaces could be affected during the removal process, adding indistinguishable bias to the results, as discussed in previous work (Yoshida et al., 2021; Zheng et al., 2022b). The encrustation volume ratio (EVR) was calculated by normalizing the encrustation volume with the corresponding stent volume, which gives the encrustation volume per stent unit. The bias introduced by the different volumes of individual stent samples was hence eliminated. The total encrustation volume ratio (TEVR) was defined by summing the EVR over the four stent sections. The end point was the encrustation risk level (ERL) on different stent sections, which was calculated by dividing EVR over TEVR. As such, the inter-subject variability between samples was removed as the ERL of each stent summed to unity (100%). The stent section with the highest ERL was interpreted as the location most susceptible to encrustations. Final results from the encrustation tests with and without the bladder model were compared and discussed.

For statistical comparisons, the two-sided Mann-Whitney *U*-test was used. *p* values from multiple comparisons were corrected using the Bonferroni-Holm method, and *p* < 0.05 was considered significant in this study.

## 3 Results

### 3.1 Pressure-flow test

**FIGURE 2:**
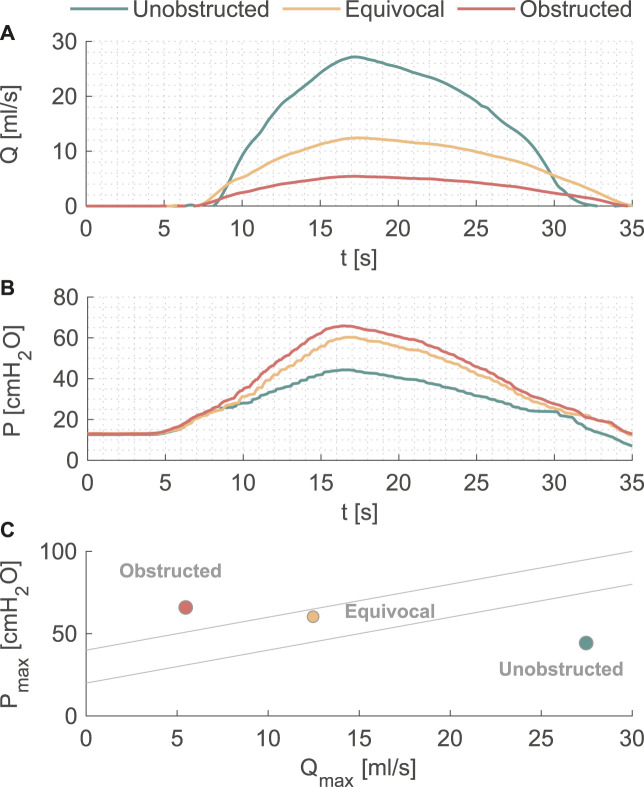
**(A)** Flow rate and **(B)** pressure variations during one voiding cycle in the *in vitro* model. **(C)** The nomogram from [12] to indicate the BOO level.

Pressure and flow rates measured during the pressure-flow tests with three outlet conditions are shown in Figures 2A,B, and further characteristics are reported in Table 1. During the filling phase, the vesical pressure was held constant at about 13 cmH_2_O. Since we imposed the same pressure waveform to initiate the voiding phase, both peak pressure and residual volume increased with higher BOO levels. The total voiding time *t* = 35 s was the same for all cases, which is within physiological range of normal urodynamic tests (Alloussi et al., 2010). The mean and peak flow rates decreased with higher BOO level as the resistance increased. According to the nomogram from (Abrams, 1999), the three test conditions covered all three categories of BOO levels (Figure 2C).

**TABLE 1:** Characteristics of the pressure-flow test at different BOO levels.

Characteristic	Unobstructed	Equivocal	Obstructed
Peak flow rate (mL/s)	27.2	12.4	5.4
Mean flow rate (mL/s)	11.7	6.3	2.7
Peak pressure (cmH_2_O)	44.3	60.3	65.9
Residual volume (mL)	69.6	259.9	383.7

### 3.2 Reflux test

**FIGURE 3:**
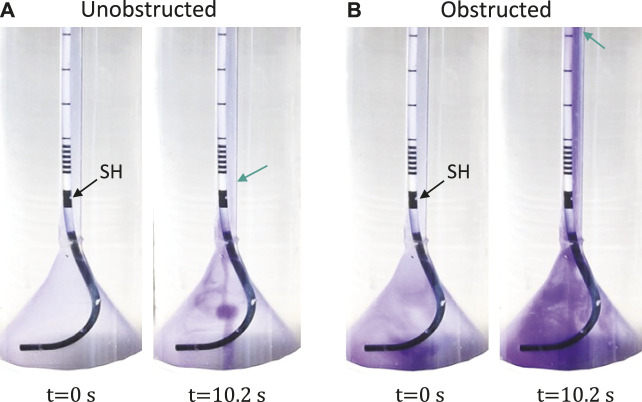
Reflux test with unobstructed **(A)** and obstructed **(B)** bladder outlet. The first SH above UVJ is marked by the black arrow. The green arrows indicate the locations of the first SH **(A)** and the mid-ureter **(B)**. Full video can be found in the Supplementary Material.

Video sequences were acquired during the reflux tests in the unobstructed (0% obstruction) and obstructed case (88% obstruction). Full videos are available in the Supplementary Material, and snapshots at *t* = 0 s and *t* = 10.2 s are given in Figure 3 for each group showing the reach of the dye color. In the unobstructed case, the retrograde reflux only arrived at the first SH above the UVJ (green arrow in Figure 3A), whereas in the obstructed case the reflux traveled beyond the mid-length of the ureter (green arrow in Figure 3B). Note that, the normal urine flow of 2 ml/min was retained during the reflux test, and the reflux did not reach up to the UPJ in either case.

### 3.3 Encrustation test

**FIGURE 4:**
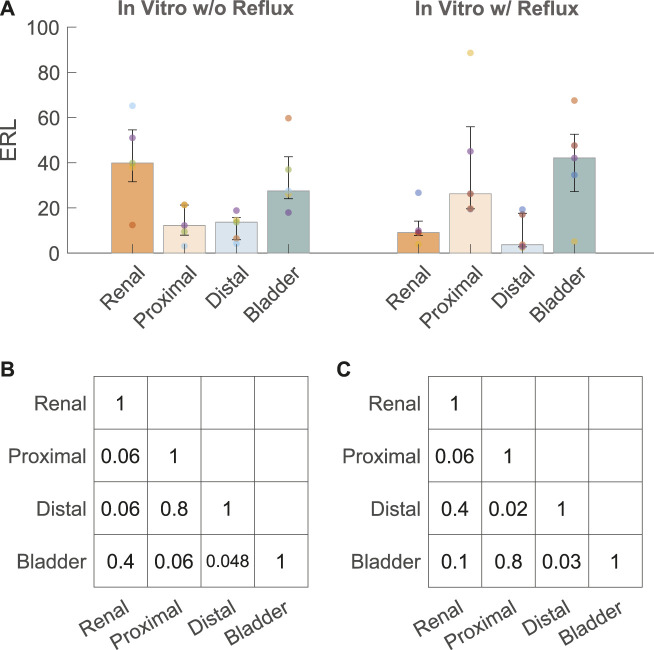
**(A)** ERL results from the *in vitro* experiments with (w/) and without (w/o) reflux. The size of the bar and error bar are given by the median and IQR, respectively. Corrected *p* values of complete comparisons between each section of the stent are given for the test group without reflux **(B)** and with reflux **(C)**.

Five stents were collected and analyzed from each experiment with and without reflux, respectively. The median and interquartile range (IQR) of the ERLs for the groups with and without reflux are given in Figure 4A. The reflux induced by the bladder model created a significant difference in the ERL between the proximal and distal straight sections (*p* = 0.02, Figure 4C), which was not observed in the group without reflux (*p* = 0.8, Figure 4B). The median ERL in the renal pigtail was lower in the group with reflux than in the group without (*p* = 0.02), and no significant difference was observed for other stent sections (Table 2).

**TABLE 2:** Median ERL (IQR) from each encrustation test group.

	ERL w/o reflux	ERL w/reflux	*p* value
Renal pigtail	39.8 (31.6–54.5)	9.1 (7.8–14.2)	0.02
Proximal	12.2 (7.9–21.3)	26.3 (19.6–55.9)	0.1
Distal	13.7 (5.9–15.7)	3.7 (2.8–17.6)	0.5
Bladder pigtail	27.5 (24.1–42.6)	42.1 (27.2–52.6)	0.5

## 4 Discussion

Truthful representation of the patho-physiological conditions *in vivo* is a challenging task for *in vitro* modeling. In an earlier study (Gorman et al., 2003) comparing dynamic and static encrustation experiments with AU, it was shown that encrustation levels on polyurethane, Percuflex, and silicone stents were all significantly higher in static test conditions. Subsequent studies often used peristaltic pump to simulate the unidirectional urine flow in various ureter models, either in full scale (Cauda et al., 2017) or in microfluidic chips (Mosayyebi et al., 2018).

However, the actual physio-mechanical condition in the upper urinary tract with an indwelling stent has two distinct phases due to the periodic bladder activity. One is during filling, which can be well represented by the aforementioned unidirectional flow with flow rates of 1–2 ml/min. The other is during voiding, which is characterized by the VUR as a result of the rising pressure induced by the bladder contraction during voiding. A realistic *in vitro* model therefore should reproduce the dynamic pressure characteristics that reflect the cyclic behaviors of the bladder.

The earliest bladder models in urological research were designed to study the bacterial growth rate against residual urine volume and the usage of antibiotics (O'Grady and Pennington, 1966; Greenwood and O'Grady, 1978). These models consisted of glass flasks with continuous incoming AU and periodic emptying. The time scale of each phase was controlled by adjusting the incoming flow rate and the interval of voiding.

Later, an encrustation model (Choong et al., 2000) of the entire upper urinary tract was proposed by assembling a small glass chamber (kidney), a glass tube (ureter), and a large glass chamber (bladder). The novelty of this setup was the siphon connected to the outlet of the bladder chamber, periodically and automatically triggering voiding when the fluid in the bladder chamber reached 300 ml. Nonetheless, the siphon could not simulate the pressure rise during voiding, and consequently the effect of VUR was not evaluated.

In another model (Kim et al., 2011), the physiological pressure condition of the bladder was modeled using a distensible rubber membrane hosted in a hemispherical capsule. The pressure condition was regulated by injecting (removing) air into (from) the space between the hemispherical capsule and the rubber membrane. A pump was used to fill the bladder with water, whereas the outflow was controlled by the air pressure and an outlet valve. Different outlet obstruction levels were simulated by partially opening the outlet valve. The proposed model (Kim et al., 2011) successfully recreated physiological pressure-volume relationships of the bladder during filling and voiding, and demonstrated the possibility to simulate physiological bladder conditions by means of mechanical modeling. At the same time, however, the complexity of the model made it difficult to be implemented in other applications.

The current study presented a simple solution to model the dynamics of the urinary bladder by connecting a pressure pump to a cylindrical vessel with a silicone tube (urethra model) as the outlet mimicking the physiological characteristics of the human urinary tract (Zheng et al., 2021). In the pressure-flow tests, we successfully reproduced pressure (Figure 2A) and flow (Figure 2B) curves corresponding to the physiological and pathological (BOO) bladder function (Figure 2C). Table 1 demonstrated the variability in flow, pressure, and residual volume achieved by simply reducing the cross-sectional area of the urethra model. Since the same voiding time (*t* = 35 s) was prescribed, the residual volume increased with increasing BOO. By re-programming the control protocol of the pressure pump and altering the physical dimensions of the components, various parameters regarding the periodic activity of the bladder can be studied, including pressure, voiding time, flow rate, and residual volume. Owing to its simple and modularized design, the model can be integrated into various testing platforms to study bacterial growth, urodynamics, or as animal model substitutes to evaluate urological devices and procedures.

In the reflux tests, the active bladder model was combined with the ureter model to visualize the VUR created in our *in vitro* setup. As shown in Figure 3A, the reflux traveled up to the first SH above UVJ when the urethra model was not obstructed. This observation is in line with a previous study in a porcine model (de la Cruz et al., 2021), showing that the stent associated VUR affected mainly the lower/distal ureter. With obstruction in the urethra, however, the dye traveled much higher than the mid-ureter, which was evidence of a stronger VUR. This perhaps calls for different management strategies for stented patients with BOO (e.g., prostate enlargement), such as choosing a stent with anti-reflux design features (Soria et al., 2018).

After examining the pressure-flow and VUR conditions, we showed that the distribution of encrustations exhibited different patterns with and without reflux (Figure 4A) using the quantification method established in an earlier study (Zheng et al., 2022b). Our results suggested that VUR might have a flushing effect on the distal section of the stent, thus removing some of the encrustations in the stent lumen, and consequently creating the observed difference between proximal and distal sections. The same difference was also observed in our previous data using stents collected from stone patients (Zheng et al., 2022b), such that a significant difference was observed in ERL between the proximal and distal sections. This might suggest that *in vitro* experiments with reflux represent the physiological conditions *in vivo* more accurately, at least in stone patients.

Clinically, proximal encrustations are often associated with difficulties removing the stent thus requiring invasive procedures (Weedin et al., 2011), yet the distribution pattern of stent encrustation remains a topic of dispute. Recently, µ-CT imaging was applied to quantify stent encrustations in clinical studies (Yoshida et al., 2021; Amitay-Rosen et al., 2022; Zheng et al., 2022b). One of them (Yoshida et al., 2021) used morphological segmentation to quantify the volume of encrustations and concluded that the entire straight part of the stent was more susceptible to luminal encrustations than the pigtails, although no observation was made to further differentiate the distribution along the straight part. In complement, another study (Amitay-Rosen et al., 2022) calculated the blockage level of the luminal area in each slice along the length of the stent (higher blockage level means more encrustation), and showed more encrustations in the proximal part of the stent near the UPJ. Both of these results agreed with our recent study using stents retrieved from stone patients (Zheng et al., 2022b). These observations might be explained by the VUR (Figures 4A,C) in the distal ureter as discussed above, assuming that VUR in stented patients is indeed common (de la Cruz et al., 2021).

By modeling the dynamic physio-mechanical characteristics of the ureter, we were able to recreate the encrustation pattern between the UPJ and UVJ, suggesting that the dynamics of urine flow, especially VUR induced by voiding, plays an important role in the encrustation process. This is supported by recent studies using microfluidic chips and numerical simulations (Mosayyebi et al., 2018; Mosayyebi et al., 2022), where the authors emphasized the role of fluid induced transport in regulating the encrustation process. Particularly, lower shear stress levels were attributed to excessive encrustations often found near the side holes (Singh et al., 2001; Sighinolfi et al., 2015; Zheng et al., 2022b; Mosayyebi et al., 2022). In this context, the current study contributed to the topic by emphasizing the role of VUR in this dynamic process. It is noted that only luminal encrustations are analyzed and discussed in this work. While we acknowledge that external encrustation is an important aspect of stent complications that are clinically relevant, reliable quantitative evaluation of the distribution of external encrustation requires three dimensional *in vivo* imaging methods with excellent resolution down to the order of 0.1 mm. At the same time, it is the side hole and the lumen of the stent that serve the crucial role of bypassing the urine flow if the extraluminal space gets obstructed (Clavica et al., 2014). Therefore, the quantitative results on luminal encrustations remain relevant. The interplay and time evolution of luminal *versus* external encrustations *in vivo* should be an interesting topic to be properly addressed in future studies.

Regarding the limitations of our results, the material properties of the urinary tract tissues were not modelled, which would affect the compliance and consequently the dynamic pressure conditions in the setup. This perhaps explains the difference (*p* = 0.02) of ERL in the renal pigtails in the encrustation test with and without reflux (Table 2). As shown in Figures 1A,B syringe was connected to the inlet of the ureter model as a compliance element in order to allow reflux during the voiding phase. As such, the flow dynamics and consequently the transport of crystalline particles in the UPJ could be different between the two encrustation tests with and without reflux. Further studies with quantitative pressure measurements at the UPJ may offer more insights into this discrepancy.

Another limitation was the connection between the ureter model and bladder model. In this current setup, the bladder pigtail was always in contact with AU in the UPJ part of the ureter model. In contrast, as a real bladder voids, at least part of the bladder pigtail will not be fully submersed in urine. How much impact this difference may have on the encrustation pattern remains unexplored. Also, if bacteria were present in the bladder, the interplay between bacterial growth and the dynamic bladder activity might be worth looking at with respect to the encrustation process.

To summarize, we designed and validated an *in vitro* bladder model to simulate both the filling and voiding phases of the urinary bladder. The pressure-flow and reflux tests demonstrated the capability of the setup to recreate patho-physiological conditions in the human urinary bladder. Our results suggested that, VUR is an important factor in regulating the dynamics in the upper urinary tract with indwelling stents, especially in patients with higher bladder outlet obstruction levels. For these patients, stents with anti-reflux features such as that proposed by Soria and colleagues (Soria et al., 2018) might be considered, where the lower part of the stent was substituted by strings to allow the closure of UVJ during voiding. Overall, we conclude that VUR is an important factor in regulating the dynamics in the upper urinary tract with indwelling stents, and that it should be properly addressed in future *in vitro* studies and clinical analyses.

## Data Availability

Data generated during the study are presented in the article and Supplementary Materials. Further inquiries can be directed to the corresponding author.
