# Supplementary material for: An in vitro bladder model with physiological dynamics: Vesicoureteral reflux alters stent encrustation pattern
Source: Front Bioeng Biotechnol. 2022 Oct 11;10:1028325. doi: 10.3389/fbioe.2022.1028325 (PMC9592728; doi:10.3389/fbioe.2022.1028325)
Supplement: Supplementary file 2 [file DataSheet1.DOCX]

Supplementary Material

An in vitro bladder model with physiological dynamics: Vesicoureteral reflux alters stent encrustation pattern

Shaokai Zheng^[[1]](#footnote-1)^ ^†1^^[[2]](#footnote-2)^⁎, Pedro Amado^†1^, Dominik Obrist^1^, Fiona Burkhard^2^, and Francesco Clavica^1, 2^

^1^ ARTORG Center for Biomedical Engineering Research, University of Bern, Bern, Switzerland

^2^ Department of Urology, Inselspital, Bern University Hospital, University of Bern, Bern, Switzerland

# Waveform for pressure pump control

The waveform used to control the pressure pump is shown in supplementary figure 1. The shape resembles a generic intravesical pressure curve during voiding, similar to that in (1, 2). By design, the pressure value used in the pump control protocol prescribes the pressure in a closed vessel. In our setup, there was an outlet (urethra model) of the bladder model. Therefore, the pressure value in supplementary figure 1 did not match the actual measurement presented in the manuscript. The discrepancy was largest for the non-obstructed case (more volume was expelled), and decreased as the bladder outlet obstruction level was increased, so that the bladder model behaved closer to a closed vessel. This waveform was defined as a series of numerical data points, which can be modified to represent various patho-physiological pressure conditions in the bladder.


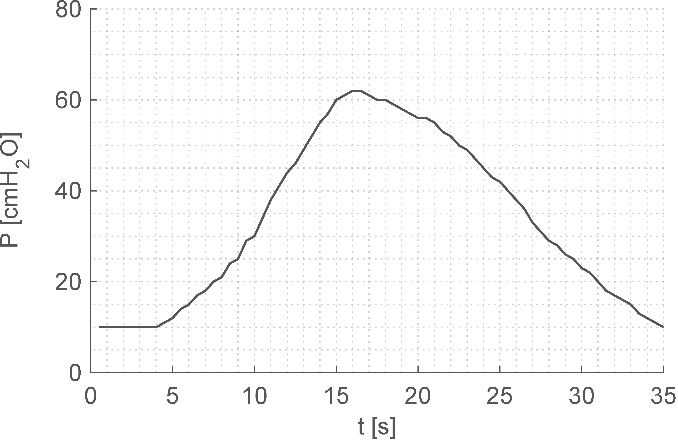


Supplementary Figure 1. Waveform of pressure for voiding control of the bladder model.

# Composition of artificial urine

The composition of the artificial urine used in this study is given in supplementary table 1, which was replicated from (3).

Supplementary Table 1. Composition of the artificial urine, reproduced from (3).

| Chemical | Quantity (g) | Concentration (mmol/L) |
| --- | --- | --- |
| Ammonium chloride | 1.34 | 25 |
| Calcium chloride dihydrate | 0.37 | 2.5 |
| Citric Acid | 0.38 | 2 |
| Lactic Acid | 0.1 | 1.1 |
| Magnesium sulfate heptahydrate | 0.49 | 2 |
| Potasium dihydrogen phosphate | 0.95 | 7 |
| Sodium bicarbonate | 2.1 | 25 |
| Sodium chloride | 5.26 | 90 |
| Sodium sulfate decahydrate | 3.22 | 10 |
| Urea | 10.21 | 170 |

# Compositions of encrustations

Here, we offer a brief assessment of the chemical compositions of the encrustations observed in this study. To do that, samples of encrusted stents were taken for Scanning Electron Microscopy (Gemini 450 SEM, Carl Zeiss AG, DE) with Energy-Dispersive X-Ray Spectroscopy (Oxford Instruments Ultim Max 65, Oxford Instruments plc, UK) at the Department of Chemistry and Biochemistry, University of Bern. The beam energy of the SEM was 20kV with working distances of 9-10mL with magnifications of 50 to 1.5K. All samples were sputter-coated with 20nm of gold to improve the signal-to-noise ratio.

Select images of the encrustations from patients and in vitro experiments are given in supplementary figure 2. Qualitatively, the encrustations were often seen in the vicinity of side holes in both cases (supplementary figure 2A and D), as reported in our preceding communication (4) and previous clinical observations (5, 6). From the patient group, calcium-based crystals such as calcium oxalate (CaC_2_O_4_, supplementary figure 2B) and calcium carbonate (CaCO_3_, supplementary figure 2C) were common. Most of the encrustations were mixed with organic substances resembling the conditioning film or biofilm. From our in vitro experiments using the artificial urine composition reported above, most frequently observed encrustation consisted of sodium chloride (NaCl, supplementary figure 2E), and calcium phosphate (Ca_3_(PO_4_)_2_}, supplementary figure 2F). The difference might be attributed to the pH environment such that calcium oxalate are often found in acidic urine environments between pH 4.5 and 5.5, whereas calcium phosphate occur between pH 6.5 and 7.5 (7, 8), which is similar to the current study condition. If the current setup were intended for chemical studies of the encrustation, the protocol of pH control need to be carefully designed.


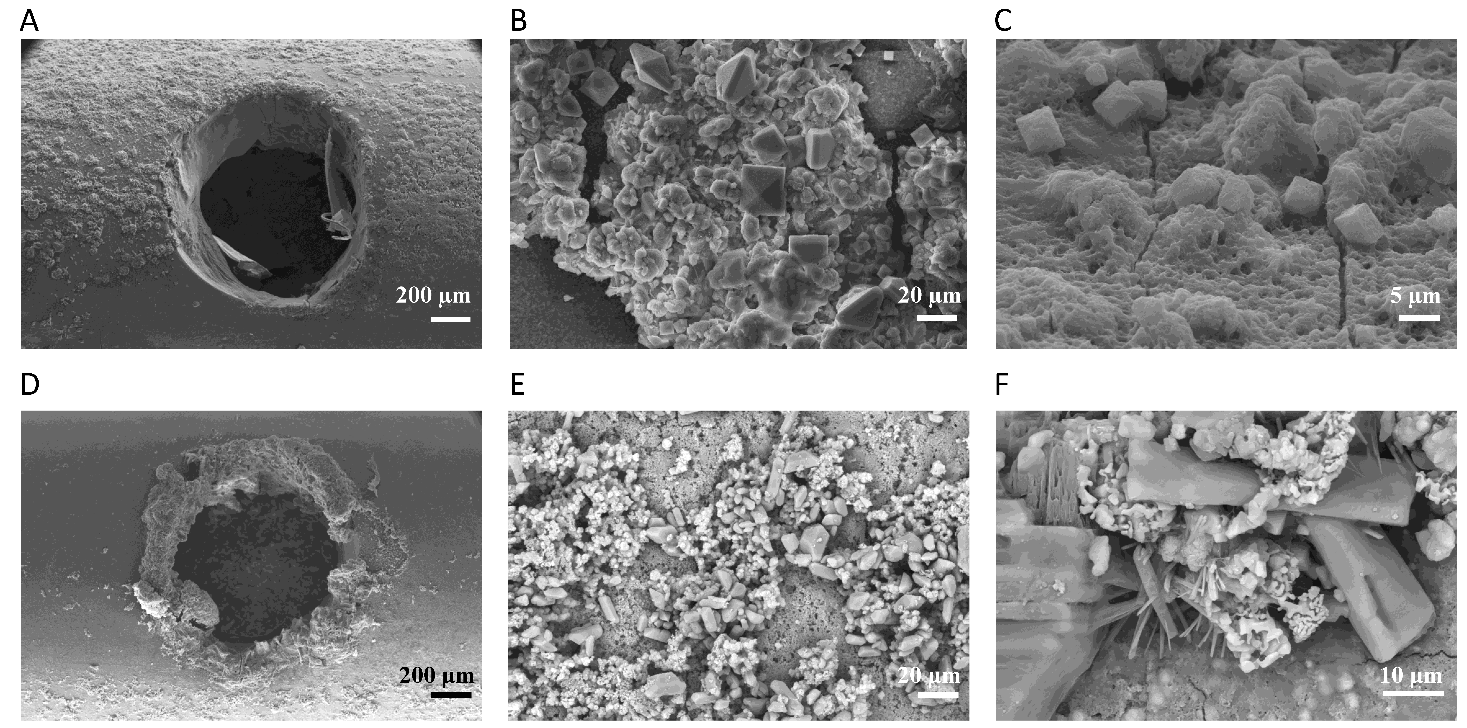


Supplementary Figure 2. Select SEM images of encrustations on stents collected from the patients (A-C) and the in vitro experiments (D-F).

References

1. Abrams P. *Urodynamics*. 3rd ed. London: Springer (2006).

2. Kim J, Lee MK, Choi B. A Study on the Fluid Mechanical Urinary Bladder Simulator and Reproduction of Human Urodynamics. *International Journal of Precision Engineering and Manufacturing* (2011) 12(4):679-85.

3. Mosayyebi A, Yue QY, Somani BK, Zhang X, Manes C, Carugo D. Particle Accumulation in Ureteral Stents Is Governed by Fluid Dynamics: In Vitro Study Using a “Stent-on-Chip” Model. *Journal of Endourology* (2018) 32(7):639-46.

4. Zheng S, Amado P, Kiss B, Stangl F, Haeberlin A, Sidler D, et al. Quantitative Evaluation of Encrustations in Double-J Ureteral Stents with Micro-Computed Tomography and Semantic Segmentation. *Frontiers in Urology* (2022) 2.

5. Singh I, Gupta NP, Hemal AK, Aron M, Seth A, Dogra PN. Severely Encrusted Polyurethane Ureteral Stents: Management and Analysis of Potential Risk Factors. *Urology* (2001) 58(4):526-31.

6. Zisman A, Siegel YI, Siegmann A, Lindner A. Spontaneous Ureteral Stent Fragmentation. *Journal of Urology* (1995) 153(3):718-21. doi: 10.1016/S0022-5347(01)67697-3.

7. Berg C, Tiselius HG. The Effect of Ph on the Risk of Calcium Oxalate Crystallization in Urine. *European Urology* (1986) 12:59-61. doi: 10.1159/000472578.

8. Manissorn J, Fong-ngern K, Peerapen P, Thongboonkerd V. Systematic Evaluation for Effects of Urine Ph on Calcium Oxalate Crystallization, Crystal-Cell Adhesion and Internalization into Renal Tubular Cells. *Scientific Reports* (2017) 7(1):1798. doi: 10.1038/s41598-017-01953-4.

1. † These authors contributed equally to this work. [↑](#footnote-ref-1)
2. ⁎ Correspondence: Shaokai Zheng, ARTORG Center for Biomedical Engineering Research, University of Bern, Freiburgstrasse 3, 3010 Bern, Switzerland. Email address: shaokai.zheng@outlook.com [↑](#footnote-ref-2)
